# Tumor-targeting *Salmonella typhimurium* A1-R inhibits human prostate cancer experimental bone metastasis in mouse models

**DOI:** 10.18632/oncotarget.5866

**Published:** 2015-09-28

**Authors:** Makoto Toneri, Shinji Miwa, Yong Zhang, Cameron Hu, Shuya Yano, Yasunori Matsumoto, Michael Bouvet, Hayao Nakanishi, Robert M. Hoffman, Ming Zhao

**Affiliations:** ^1^ AntiCancer, Inc., San Diego, California, USA; ^2^ Department of Surgery, University of California, San Diego, California, USA; ^3^ Ageo Medical Group, Saitama, Japan; ^4^ Laboratory of Pathology and Clinical Research, Aichi Cancer Center Aichi Hospital, Aichi, Japan

**Keywords:** prostate cancer, bone metastasis, GFP/RFP, zoledronic acid, bacterial therapy

## Abstract

Bone metastasis is a frequent occurrence in prostate cancer patients and often is lethal. Zoledronic acid (ZOL) is often used for bone metastasis with limited efficacy. More effective models and treatment methods are required to improve the outcome of prostate cancer patients. In the present study, the effects of tumor-targeting *Salmonella typhimurium* A1-R were analyzed *in vitro* and *in vivo* on prostate cancer cells and experimental bone metastasis. Both ZOL and *S. typhimurium* A1-R inhibited the growth of PC-3 cells expressing red fluorescent protien *in vitro*. To investigate the efficacy of *S. typhimurium* A1-R on prostate cancer experimental bone metastasis, we established models of both early and advanced stage bone metastasis. The mice were treated with ZOL, *S. typhimurium* A1-R, and combination therapy of both ZOL and *S. typhimurium* A1-R. ZOL and *S. typhimurium* A1-R inhibited the growth of solitary bone metastases. *S. typhimurium* A1-R treatment significantly decreased bone metastasis and delayed the appearance of PC-3 bone metastases of multiple mouse models. Additionally, *S. typhimurium* A1-R treatment significantly improved the overall survival of the mice with multiple bone metastases. The results of the present study indicate that *S. typhimurium* A1-R is useful to prevent and inhibit prostate cancer bone metastasis and has potential for future clinical use in the adjuvant setting.

## INTRODUCTION

Prostate cancer is diagnosed in more than 500,000 men worldwide [1]. Bone metastases from prostate cancer can cause chronic pain, hypercalcemia, pathologic fractures, and nerve compression [2]. Zoledronic acid (ZOL), a bisphosphonate, has been used to prevent the development of metastatic bone lesions. Although bisphosphonates, irradiation, and surgical resection of tumors are used as treatments of bone metastases, the outcomes of the treatments are still unsatisfactory.

*Salmonella typhimuium* (*S. typhimurium*) A1-R, is a genetically-engineered strain of *Salmonella* that is able to specifically target cancer cells [3]. *S. typhimurium* A1-R was able to inhibit or eradicate primary and metastatic tumors as monotherapy in nude mouse models of prostate [3, 4], breast [5–7], lung [8, 9], pancreatic [10–14], ovarian [15, 16], stomach [17] and cervical cancer [18], as well as sarcoma [19–23] and glioma [24, 25], all of which are highly aggressive tumor models. Tumors with a high degree of vascularity were more sensitive to *S. typhimurium* A1-R, and vascular destruction appears to play a role in *S. typhimurium* A1-R antitumor efficacy [25]. Tumor vessel destruction and tumor-growth inhibition were enhanced by primer dosing of *S. typhimurium* A1-R in immunocompetent transgenic mice expressing the nestin-driven green fluorescent protein (ND-GFP), which is selectively expressed in nascent blood vessels [26].

In the present study, the efficacy of *S. typhimurium* A1-R and the combination of *S. typhimurium* A1-R and ZOL was assessed in nude mice models of solitary and multiple bone metastases of prostate cancer.

## RESULTS AND DISCUSSION

### Efficacy of *S. typhimurium* A1-R on human prostate cancer cells *in vitro*

**Figure 1:**
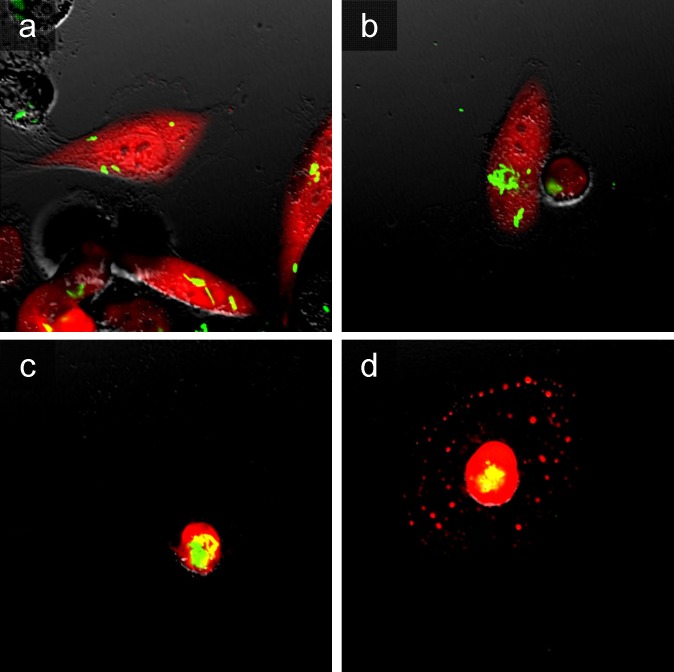
Efficacy of *S. typhimurium* A1-R *in vitro* on prostate cancer cells PC-3-RFP cells were incubated in 35 mm dishes for 24 h. The cells were treated with *S. typhimurium* A1-R for 1 h. The PC-3-RFP cells were rinsed with PBS and the cells were observed with the FV1000 confocal microscope. Fluorescence images were obtained at 24 hours after infection and demonstrated that *S. typhimurium* A1-R expressing GFP invaded **a.** and replicated intracellularly **b.** in PC-3-RFP cells. After the invasion and replication of GFP-expressing *S. typhimuium* A1-R, the infected cells shrunk **c.** and fragmented **d.** See Materials and Methods for details.

**Figure 2:**
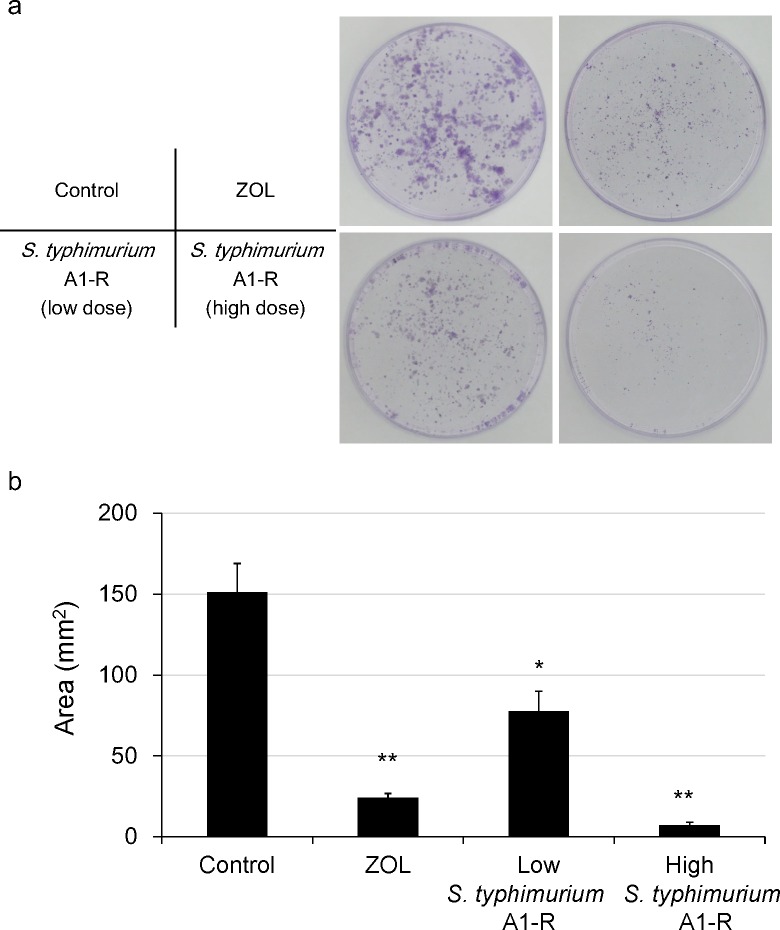
Growth inhibitory effect of A1-R *in vitro* Clonogenic assays were performed as previously described [40]. PC-3-RFP cells 1 × 10^3^ were incubated in 35 mm dishes. PC-3-RFP colonies were fixed with methanol and stained with 1% crystal violet 8 days after zoledronic acid (ZOL) or *S. typhimuium* A1-R (1 × 10^7^ or 1 × 10^8^ CFU/dish). PC-3-RFP cells were plated in 35 mm dishes. ImageJ (National Institutes of Health, Bethesda, Maryland, USA) was used to quantify the areas of the colonies of the cells. **a.** Colonies after treatment with ZOL or *S. typhimuium* A1-R. **b.** PC-3-RFP colony area decreased after treatment with ZOL or *S. typhimuium* A1-R. ZOL inhibited the growth of PC-3-RFP cells compared to the untreated control group. *S. typhimuium* A1-R also inhibited the growth of PC-3-RFP cells in a dose-dependent manner (*p < 0.05, **p < 0.01).

To determine the efficacy of A1-R on prostate cancer cells, PC-3-RFP cells were incubated in 35 mm dishes for 24 h, and the cells were treated with *S. typhimurium* A1-R for 1 h. The cells were observed with a Fluoview FV1000 confocal microscope (Olympus Corp., Tokyo, Japan). Fluorescence imaging demonstrated that *S. typhimurium* A1-R expressing GFP selectively invaded and replicated intracellularly and killed PC-3-RFP cells (Figure 1). Clonogenic assays demonstrated that *S. typhimurium* A1-R inhibited proliferation of PC-3-RFP cells in a dose-dependent manner (Figure 2).

### Efficacy of *S. typhimurium* A1-R therapy on a mouse model of multiple bone metastasis

**Figure 3:**
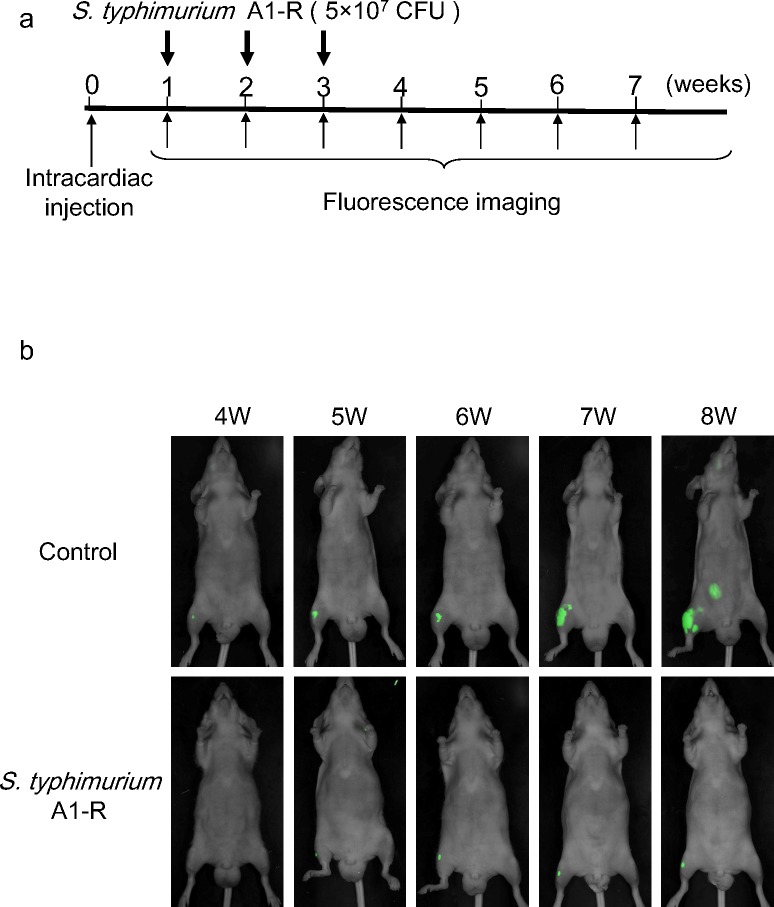

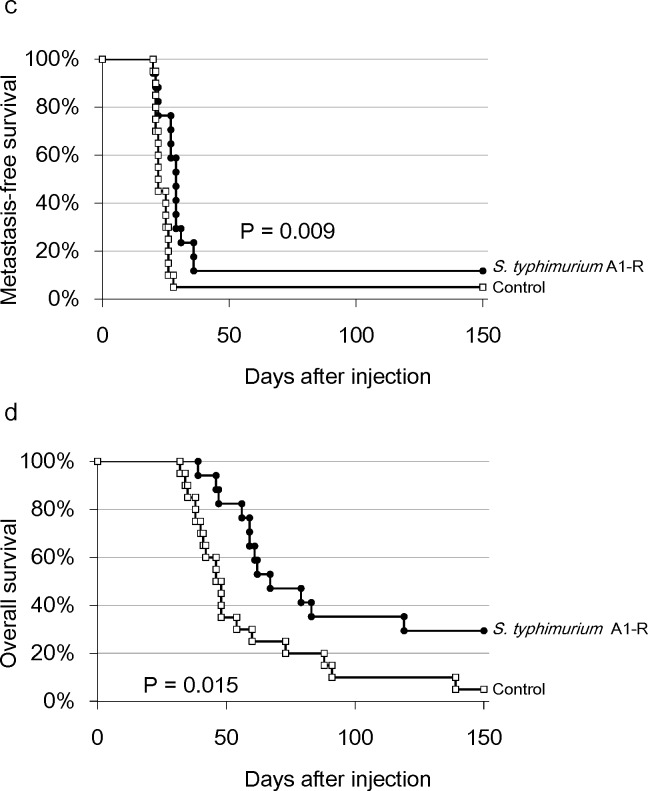
Efficacy of *S. typhimurium* A1-R on multiple bone metastasis **a.** PC-3-GFP cells were injected into the left ventricle in nude mice. On day 7, 14, and 21, *S. typhimurium* A1-R (5×10^7^ CFU/mouse) was administered i.v. Fluorescence imaging was performed every week. Metastasis-free survival and overall survival of mice treated with *S. typhimurium* A1-R or untreated controls was determined using the Kaplan-Meier method with the log-rank test. **b.** Time-course fluorescence imaging of prostate cancer bone metastasis with or without treatment of *S. typhimuium* A1-R. PC-3-GFP cells were inoculated into the left cardiac ventricle of nude mice. Fluorescence imaging visualized the progression of metastases in control mice. In contrast, there was only a small increase in fluorescence in the mice treated with *S. typhimuium* A1-R. **c.** Metastasis-free survival. **d.** Overall survival.

Nude mice were injected in the left ventricle with PC-3-GFP cells (5 × 10^5^). One week after intracardiac injection, half of the mice were treated once a week for 3 weeks with an i.v. injection of *S. typhimurium* A1-R (5 × 10^7^ CFU) (Figure 3a). Time-course fluorescence imaging of the study mice revealed GFP-expressing tumor growth in the control group and little tumor growth in the *S. typhimurium* A1-R group (Figure 3b). *S. typhimurium* A1-R significantly improved the metastasis-free survival and overall survival of the mice (Figure 3c, 3d).

### Efficacy of *S. typhimurium* A1-R on a mouse model of solitary bone metastasis of prostate cancer

**Figure 4:**
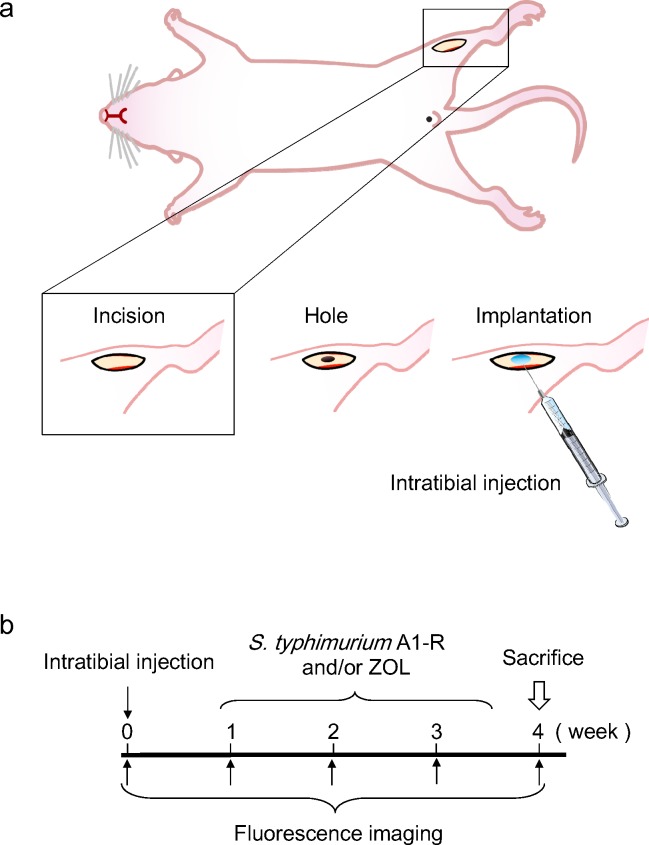

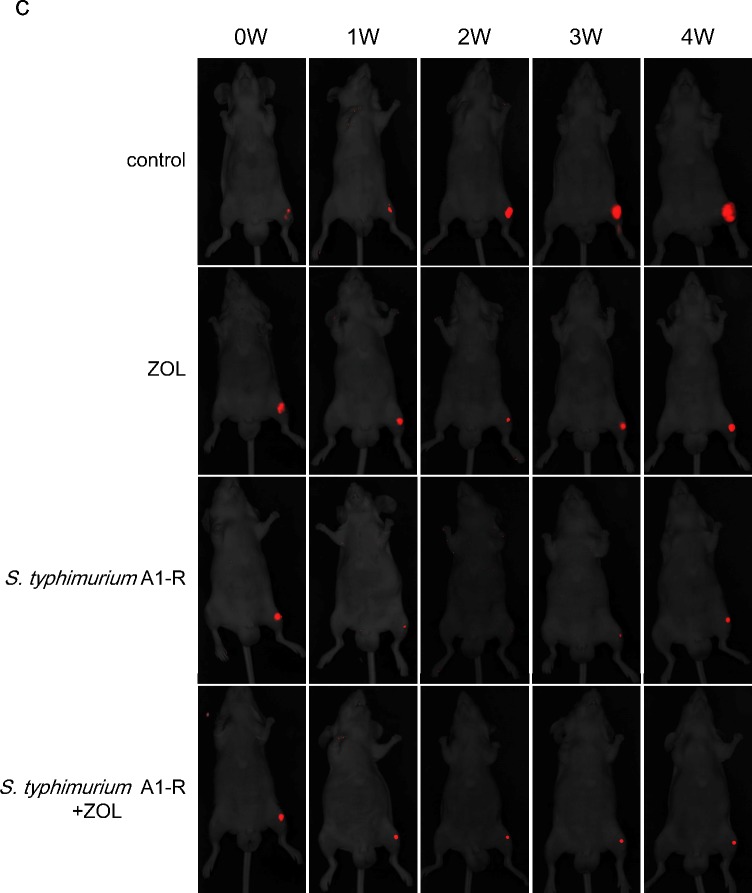

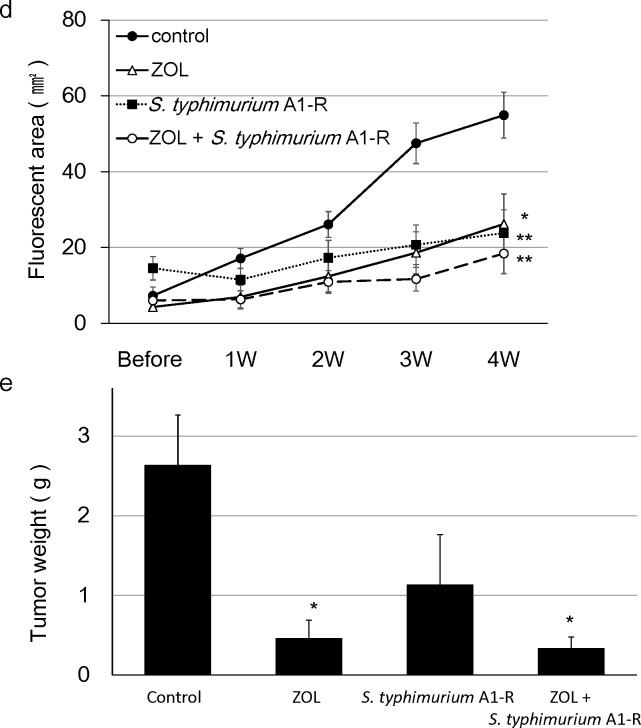
Efficacy of *S. typhimurium* A1-R on solitary bone metastasis **a.** A 5 mm midline skin incision was made to expose the tibial tuberosity. PC-3-RFP cells (5 × 10^5^) in Matrigel (5 μl) (BD Bioscience, San Jose, CA) were injected in the intramedullary cavity of the tibia. **b.** One week after intratibial injection, fluorescence imaging was performed to confirm the growing RFP-expressing tumor using the iBOX Scientia Small Animal Imaging System (UVP LLC, Upland, CA, USA). The *S. typhimuium* A1-R group was administered *S. typhimurium* A1-R (5 × 10^7^ CFU, i.v.) once a week for 3 weeks. The control group was administered the same volume of PBS. Fluorescence imaging was performed on treated and untreated mice. RFP fluorescent area was recorded every week for 5 weeks using the iBOX. **c.** Time-course imaging of the RFP-expressing bone tumors in the mouse model of solitary bone metastasis after treatment with ZOL and *S. typhimuium* A1-R. **d.** Fluorescence area of bone tumors in the control group and A1-R group mice. *p < 0.05, ** *p* < 0.01 compared with the control group. e. Tumor weight at the end of the experiment in mice injected intratibially with PC-3-RFP cells. Data are expressed as the mean ± SE. Differences between groups were analyzed with ANOVA. * *p* < 0.05 compared with the untreated control group.

PC-3-RFP (5 × 10^5^) cells were injected into the intramedullary cavity of the tibia in nude mice (Figure 4a). One week after the injection, the mice were divided into 4 groups: a control group, a ZOL group, an A1-R group, and a ZOL+ *S. typhimurium* A1-R group. ZOL-group mice were treated with subcutaneous injection of ZOL (120 mg/kg) (Sigma-Aldrich. St. Louis, MO) 5 times a week for 4 weeks (Figure 4b). *S. typhimurium* A1-R group mice were treated with weekly i.v. injections of *S. typhimurium* A1-R (5 × 10^7^ CFU) for a total of 3 weeks. ZOL+ *S. typhimurium* A1-R-group mice were treated with both s.c. injection of ZOL and i.v. injection of *S. typhimurium* A1-R at the same dosages listed above. Fluorescence imaging was performed with an iBOX Scientia Imaging System (UVP, LLC, Upland, CA) every week. The fluorescence images demonstrated that the control group had rapid growth of metastatic bone cancer, whereas the ZOL group, *S. typhimurium* A1-R group, and the ZOL+*S. typhimurium* A1-R group mice had reduced metastatic growth (Figure 4c). The fluorescent tumor area of the control-group mice was 54.9 ± 6.1 mm^2^, ZOL group at 4 weeks mice was 29.1 ± 9.2 mm^2^, *S. typhimurium* A1-R-group mice was 23.8 ± 6.1 mm^2^, and ZOL+ *S. typhimurium* A1-R group mice was 18.4 ± 5.3 mm^2^ (Figure 4d). The *S. typhimurium* A1-R group and the ZOL+ *S. typhimurium* A1-R group had significant inhibition of tumor growth compared to the control group. Four weeks after the injection of cancer cells, the mice were sacrificed and tumor weight was measured. Tumor weights of the control group was 2.6 ± 0.6 g; ZOL group was 0.5 ± 0.2 g; *S. typhimurium* A1-R group was 1.1 ± 0.6; and ZOL+ *S. typhimurium* A1-R group mice at 4 weeks was 0.3 ± 0.1 g (Figure 4e).

The tumor targeting of prostate cancer-bone metastasis strategy, developed in the present report, could also be used with previously-developed tumor targeting strategies [27–34].

The present study demonstrates that *S. typhimuium* A1-R could significantly inhibit or prevent prostate cancer metastasis in the bone. These results indicate a promising approach to a currently highly treatment-resistant disease.

## MATERIALS AND METHODS

### Cell culture

PC-3 cells, which were originally established from metastatic bone lesions of human prostate cancer [35], were labeled with GFP or RFP. PC-3-RFP and PC-3-GFP cells were generated as previously described [36–39]. Cells were maintained in RPMI 1640 with 10% FBS + 1% penicillin streptomycin solution. Cells were subcultured for at least 3 passages before harvesting at their linear growth phase (approximately 70-80% confluent) for intracardiac or intramedullaly injection at 5 × 10^5^ cells/mouse.

### Animal care

Athymic nude mice (nu/nu) (AntiCancer Inc, San Diego, CA) were used in this study. Mice were maintained in a barrier facility of high efficiency particulate air-filtered racks. The animals were fed an autoclaved laboratory rodent diet. Animal experiments were performed in accordance with the Guidelines for the Care and Use of Laboratory Animals under National Institutes of Health assurance number A3873-01.

### Preparation of *S. typhimurium* A1-R

GFP-expressing *S. typhimurium* A1-R bacteria (AntiCancer Inc., San Diego, CA, USA) were grown overnight on LB medium (Fisher Sci., Hanover Park, IL, USA) and then diluted 1:10 in LB medium. Bacteria were harvested at late-log phase, washed with PBS, and then diluted in PBS [3, 5].

### *S. typhimurium* A1-R killing of prostate cancer cells *in vitro*

PC-3-RFP cells were plated in 35 mm dishes (5 × 10^2^ cells/dish). *S. typhimurium* A1-R-GFP was added to the cancer cells (1 × 10^7^ or 1 × 10^8^ CFU/dish). After 1 h incubation at 37°C, the cells were rinsed and cultured in medium containing gentamycin sulfate (100 μg/ml) to kill external but not internal bacteria. Invasion and destruction of PC-3-RFP cells by *S. typhimurium* A1-R-GFP was visualized with a Fluoview FV1000 confocal microscope (Olympus Corp., Tokyo, Japan). Eight days after treatment with *S. typhimurium* A1-R, PC-3-RFP colonies were fixed in methanol and stained with 1% crystal violet as previously described [40]. ImageJ (National Institute of Mental Health, Bethesda, Maryland, USA) was used to quantify the area of the colonies of the cells.

### Efficacy of *S. typhimurium* A1-R therapy on a mouse model of multiple bone metastases

PC-3-GFP cells (5 × 10^5^) were injected intracardially in nude mice. One week after injection, mice (treatment group) were administered *S. typhimurium* A1-R (5 × 10^7^ CFU, i.v.) once a week for 3 weeks. The remaining mice (control group) were administered the same volume of PBS. To evaluate metastasis-free survival, GFP-expressing lesions were initially observed using the Illumatool imaging system(Lighttools Research, Encinitas, CA) every 2 days. Metastasis-free survival was defined as the time from intracardiac injection of cancer cells to the time of detection of bone metastases with the Illumatool.

### Efficacy of *S. typhimurium* therapy on a mouse model of solitary bone metastases

A midline skin incision (5 mm) was made just below the knee joint to expose the tibial tuberosity (Figure 4a). Matrigel (5 μL) (BD Bioscience, San Jose, CA) and PC-3-RFP cells (5 × 10^5^) were co-injected into the intramedullary cavity of the tibia with a 1.0 mL 28 G latex-free insulin syringe (BD and Company, Franklin Lakes, NJ). The skin was closed with a 6-0 suture. One week after injection, fluorescence imaging was performed to confirm the RFP-expressing tumor was growing, using the iBOX Scientia (UVP LLC, Upland, CA). The study mice were randomly divided into the following groups: control group; ZOL group; *S. typhimuium* A1-R group; and ZOL+ *S. typhimuium* A1-R-group. In the ZOL group, mice were treated with subcutaneous injection of 120 mg/kg ZOL for 5 times a week for 4 weeks (Figure 4b). *S. typhimuium* A1-R-group mice were administered *S. typhimurium* A1-R (5 × 10^7^ CFU, i.v.) once a week for 3 weeks. ZOL+ *S. typhimuium* A1-R-group mice were administered both treatments. Fluorescence imaging was performed on treated and untreated mice, and GFP-expressing areas were recorded every week for 5 weeks using the iBOX. At the end of the follow-up, the mice were sacrificed and the metastatic tumors were excised. Tumor weight was compared to evaluate the efficacy of ZOL and *S. typhimuium* A1-R.

### Statistical analysis

Data showing comparisons among 3 or more groups were assessed using analysis of variance (ANOVA). The Kaplan-Meier method was used for bone metastasis-free survival and overall survival. Log-rank tests were used for statistical significance of the difference between the two groups. Differences were considered significant when *p* < 0.05. Data are expressed as mean ± SE. Statistical analyses were performed with EZR (Saitama Medical Center, Jichi Medical University).

## References

[1] Grönberg H (2003). Prostate cancer epidemiology. Lancet.

[2] Gralow JR, Biermann JS, Farooki A, Fornier MN, Gagel RF, Kumar RN, Shapiro CL, Shields A, Smith MR, Srinivas S, Van Poznak CH (2009). NCCN Task Force Report: Bone Health in Cancer Care. J Natl Compr Canc Netw.

[3] Zhao M, Yang M, Li XM, Jiang P, Baranov E, Li S, Xu M, Penman S, Hoffman RM (2005). Tumor-targeting bacterial therapy with amino acid auxotrophs of GFP-expressing Salmonella typhimurium. Proc Natl Acad Sci USA.

[4] Zhao M, Geller J, Ma H, Yang M, Penman S, Hoffman RM (2007). Monotherapy with a tumor-targeting mutant of Salmonella typhimurium cures orthotopic metastatic mouse models of human prostate cancer. Proc Natl Acad Sci USA.

[5] Zhao M, Yang M, Ma H, Li X, Tan X, Li S, Yang Z, Hoffman RM (2006). Targeted therapy with a Salmonella typhimurium leucine-arginine auxotroph cures orthotopic human breast tumors in nude mice. Cancer Res.

[6] Zhang Y, Tome Y, Suetsugu A, Zhang L, Zhang N, Hoffman RM, Zhao M (2012). Determination of the optimal route of administration of Salmonella typhimurium A1-R to target breast cancer in nude mice. Anticancer Res.

[7] Zhang Y, Miwa S, Zhang N, Hoffman RM, Zhao M (2015). Tumor-targeting Salmonella typhimurium A1-R arrests growth of breast-cancer brain metastasis. Oncotarget.

[8] Uchugonova A, Zhao M, Zhang Y, Weinigel M, König K, Hoffman RM (2012). Cancer-cell killing by engineered Salmonella imaged by multiphoton tomography in live mice. Anticancer Res.

[9] Liu F, Zhang L, Hoffman RM, Zhao M (2010). Vessel destruction by tumor-targeting Salmonella typhimurium A1-R is enhanced by high tumor vascularity. Cell Cycle.

[10] Nagakura C, Hayashi K, Zhao M, Yamauchi K, Yamamoto N, Tsuchiya H, Tomita K, Bouvet M, Hoffman RM (2009). Efficacy of a genetically-modified Salmonella typhimurium in an orthotopic human pancreatic cancer in nude mice. Anticancer Res.

[11] Yam C, Zhao M, Hayashi K, Ma H, Kishimoto H, McElroy M, Bouvet M, Hoffman RM (2010). Monotherapy with a tumor-targeting mutant of S. typhimurium inhibits liver metastasis in a mouse model of pancreatic cancer. J Surg Res.

[12] Hiroshima Y, Zhao M, Zhang Y, Maawy A, Hassanein MK, Uehara F, Miwa S, Yano S, Momiyama M, Suetsugu A, Chishima T, Tanaka K, Bouvet M, Endo I, Hoffman RM (2013). Comparison of efficacy of Salmonella typhimurium A1-R and chemotherapy on stem-like and non-stem human pancreatic cancer cells. Cell Cycle.

[13] Hiroshima Y, Zhao M, Maawy A, Zhang Y, Katz MH, Fleming JB, Uehara F, Miwa S, Yano S, Momiyama M, Suetsugu A, Chishima T, Tanaka K, Bouvet M, Endo I, Hoffman RM (2014). Efficacy of Salmonella typhimurium A1-R versus chemotherapy on a pancreatic cancer patient-derived orthotopic xenograft (PDOX). J Cell Biochem.

[14] Hiroshima Y, Zhang Y, Murakami T, Maawy AA, Miwa S, Yamamoto M, Yano S, Sato S, Momiyama M, Mori R, Matsuyama R, Chishima T, Tanaka K, Ichikawa Y, Bouvet M, Endo I, Zhao M, Hoffman RM (2014). Efficacy of tumor-targeting Salmonella typhimurium A1-R in combination with anti-angiogenesis therapy on a pancreatic cancer patient-derived orthotopic xenograph (PDOX) and cell line mouse models. Oncotarget.

[15] Matsumoto Y, Miwa S, Zhang Y, Hiroshima Y, Yano S, Uehara F, Yamamoto M, Toneri M, Bouvet M, Matsubara H, Hoffman RM, Zhao M (2014). Efficacy of tumor-targeting Salmonella typhimurium A1-R on nude mouse models of metastatic and disseminated human ovarian cancer. J Cell Biochem.

[16] Matsumoto Y, Miwa S, Zhang Y, Zhao M, Yano S, Uehara F, Yamamoto M, Hiroshima Y, Toneri M, Bouvet M, Matsubara H, Tsuchiya H, Hoffman RM (2015). Intraperitoneal administration of tumor-targeting Salmonella typhimurium A1-R inhibits disseminated human ovarian cancer and extends survival in nude mice. Oncotarget.

[17] Yano S, Zhang Y, Zhao M, Hiroshima Y, Miwa S, Uehara F, Kishimoto H, Tazawa H, Bouvet M, Fujiwara T, Hoffman RM (2014). Tumor-targeting Salmonella typhimurium A1-R decoys quiescent cancer cells to cycle as visualized by FUCCI imaging and become sensitive to chemotherapy. Cell Cycle.

[18] Hiroshima Y, Zhang Y, Zhao M, Zhang N, Murakami T, Maawy A, Mii S, Uehara F, Yamamoto M, Miwa S, Yano S, Momiyama M, Mori R, Matsuyama R, Chishima T, Tanaka K, Ichikawa Y, Bouvet M, Endo I, Hoffman RM (2015). Tumor-targeting Salmonella typhimurium A1-R in combination with Trastuzumab eradicates HER-2-positive cervical cancer cells in patient-derived mouse models. PLoS One.

[19] Hayashi K, Zhao M, Yamauchi K, Yamamoto N, Tsuchiya H, Tomita K, Hoffman RM (2009). Cancer metastasis directly eradicated by targeted therapy with a modified Salmonella typhimurium. J Cell Biochem.

[20] Hayashi K, Zhao M, Yamauchi K, Yamamoto N, Tsuchiya H, Tomita K, Kishimoto H, Bouvet M, Hoffman RM (2009). Systemic targeting of primary bone tumor and lung metastasis of high-grade osteosarcoma in nude mice with a tumor-selective strain of Salmonella typhimurium. Cell Cycle.

[21] Tome Y, Zhang Y, Momiyama M, Maehara H, Kanaya F, Tomita K, Tsuchiya H, Bouvet M, Hoffman RM, Zhao M (2013). Primer dosing of S. typhimurium A1-R potentiates tumor-targeting and efficacy in immunocompetent mice. Anticancer Res.

[22] Miwa S, Zhang Y, Baek K-E, Uehara F, Yano S, Yamamoto M, Hiroshima Y, Matsumoto Y, Kimura H, Hayashi K, Yamamoto N, Tsuchiya H, Hoffman RM, Zhao M (2014). Inhibition of spontaneous and experimental lung metastasis of soft-tissue sarcoma by tumor-targeting Salmonella typhimurium A1-R. Oncotarget.

[23] Hiroshima Y, Zhao M, Zhnag N, Maawy A, Murakami T, Mii S, Uehara F, Yamamoto M, Miwa S, Yano S, Momiyama M, Mori R, Matsuyama R, Chishima T, Tanaka K, Ichikawa Y, Bouvet M, Endo I, Hoffman RM (2015). Tumor-targeting Salmonella typhimurium A1-R arrests a chemo-resistant patient soft-issue sarcoma in nude mice. PLoS One.

[24] Kimura H, Zhang L, Zhao M, Hayashi K, Tsuchiya H, Tomita K, Bouvet M, Wessels J, Hoffman RM (2010). Targeted therapy of spinal cord glioma with a genetically-modified Salmonella typhimurium. Cell Proliferation.

[25] Momiyama M, Zhao M, Kimura H, Tran B, Chishima T, Bouvet M, Endo I, Hoffman RM (2012). Inhibition and eradication of human glioma with tumor-targeting Salmonella typhimurium in an orthotopic nude-mouse model. Cell Cycle.

[26] Liu F, Zhang L, Hoffman RM, Zhao M (2010). Vessel destruction by tumor-targeting Salmonella typhimurium A1-R is enhanced by high tumor vascularity. Cell Cycle.

[27] Blagosklonny MV (2005). How cancer could be cured by 2015. Cell Cycle.

[28] Blagosklonny MV (2003). Tissue-selective therapy of cancer. Br J Cancer.

[29] Blagosklonny MV (2003). Matching targets for selective cancer therapy. Drug Discov Today.

[30] Blagosklonny MV (2008). “Targeting the absence” and therapeutic engineering for cancer therapy. Cell Cycle.

[31] Blagosklonny MV (2005). Teratogens as anti-cancer drugs. Cell Cycle.

[32] Blagosklonny MV (2001). Treatment with inhibitors of caspases, that are substrates of drug transporters, selectively permits chemotherapy-induced apoptosis in multidrug-resistant cells but protects normal cells. Leukemia.

[33] Blagosklonny MV (2006). Target for cancer therapy: proliferating cells or stem cells. Leukemia.

[34] Blagosklonny MV (2007). Cancer stem cell and cancer stemloids: from biology to therapy. Cancer Biol Ther.

[35] Kaighn ME, Narayan KS, Ohnuki Y, Lechner JF, Jones LW (1979 Jul). Establishment and characterization of a human prostatic carcinoma cell line (PC-3). Invest Urol.

[36] Yang M, Jiang P, Sun FX, Hasegawa S, Baranov E, Chishima T, Shimada H, Moossa AR, Hoffman RM (1999). A fluorescent orthotopic bone metastasis model of human prostate cancer. Cancer Res.

[37] Hoffman RM, Yang M (2006). Subcellular imaging in the live mouse. Nature Protocols.

[38] Hoffman RM, Yang M (2006). Color-coded fluorescence imaging of tumor-host interactions. Nature Protocols.

[39] Hoffman RM, Yang M (2006). Whole-body imaging with fluorescent proteins. Nature Protocols.

[40] Miwa S, Yano S, Tome Y, Sugimoto N, Hiroshima Y, Uehara F, Mii S, Kimura H, Hayashi K, Efimova EV, Fujiwara T, Tsuchiya H, Hoffman RM (2013). Dynamic color-coded fluorescence imaging of the cell-cycle phase, mitosis, and apoptosis demonstrates how caffeine modulates cisplatinum efficacy. J Cell Biochem.

